# Integrating virtual screening, pharmacoinformatics profiling, and molecular dynamics: identification of promising inhibitors targeting 3CLpro of SARS-CoV-2

**DOI:** 10.3389/fmolb.2023.1306179

**Published:** 2024-03-07

**Authors:** Abeer Mohammad, Ahmed Zheoat, Amjad Oraibi, Ajay Manaithiya, Khalid S. Almaary, Hiba Allah Nafidi, Mohammed Bourhia, Soumaya Kilani-Jaziri, Yousef A. Bin Jardan

**Affiliations:** ^1^ Department of Pharmacy, Al-Manara College for Medical Sciences, Maysan, Iraq; ^2^ Advanced Medical and Dental Institute, University Sains Malaysia, Kepala Batas, Pulau, Penang, Malaysia; ^3^ Department of Pharmaceutical Sciences A, Faculty of Pharmacy of Monastir, University of Monastir, Monastir, Tunisia; ^4^ Research Unit for Bioactive Natural Products and Biotechnology UR17ES49, Faculty of Dental Medicine of Monastir, University of Monastir, Monastir, Tunisia; ^5^ Department of Medicinal Chemistry, Jamia Hamdard, New Delhi, India; ^6^ Department of Botany and Microbiology, College of Science, King Saud University, Riyadh, Saudi Arabia; ^7^ Department of Food Science, Faculty of Agricultural and Food Sciences, Laval University, Quebec, QC, Canada; ^8^ Department of Chemistry and Biochemistry, Faculty of Medicine and Pharmacy, Ibn Zohr University, Laayoune, Morocco; ^9^ Department of Pharmaceutics, College of Pharmacy, King Saud University, Riyadh, Saudi Arabia

**Keywords:** SARS-CoV-2, 3CLpro, virtual screening, admet, molecular docking, molecular dynamics

## Abstract

**Introduction:** The pursuit of effective therapeutic solutions for SARS-CoV-2 infections and COVID-19 necessitates the repurposing of existing compounds. This study focuses on the detailed examination of the central protease, 3-chymotrypsin-like protease (3CLpro), a pivotal player in virus replication. The combined approach of molecular dynamics simulations and virtual screening is employed to identify potential inhibitors targeting 3CLpro.

**Methods:** A comprehensive virtual screening of 7120 compounds sourced from diverse databases was conducted. Four promising inhibitors, namely **EN1036**, **F6548-4084**, **F6548-1613**, and **PUBT44123754**, were identified. These compounds exhibited notable attributes, including high binding affinity (ranging from −5.003 to −5.772 Kcal/mol) and superior Induced Fit Docking scores (ranging from −671.66 to −675.26 Kcal/mol) compared to co-crystallized ligands.

**Results:** In-depth analysis revealed that **F6548-1613** stood out, demonstrating stable hydrogen bonds with amino acids His41 and Thr62. Notably, **F6548-1613** recorded a binding energy of −65.72 kcal/mol in Molecular Mechanics Generalized Born Surface Area (MMGBSA) simulations. These findings were supported by Molecular Dynamics simulations, highlighting the compound’s efficacy in inhibiting 3CLpro.

**Discussion:** The identified compounds, in compliance with Lipinski’s rule of five and exhibiting functional molecular interactions with 3CLpro, present promising therapeutic prospects. The integration of *in silico* methodologies significantly expedites drug discovery, laying the foundation for subsequent experimental validation and optimization. This approach holds the potential to develop effective therapeutics for SARS-CoV-2.

## 1 Introduction

A new virus known as SARS-CoV-2 emerged and was responsible for the global COVID-19 pandemic, prompting unprecedented scientific efforts to discover possible therapeutic substances and develop innovative interventions to combat this virus (Zhu et al., 2020). SARS-CoV-2’s replication and transcription cycle are influenced significantly by the 3C-like protease (3CLpro), also called the main protease (Mpro). Following the entry of viral RNA into the host cell, this protease participates in the proteolytic processing of viral polyproteins and initially produces two large precursor polyproteins, pp1a and pp1ab (Jin et al., 2020; Manaithiya et al., 2023).

For the polyproteins PP1a and PP1ab to perform different functions in the replication and transcription of the virus, they need to be cleaved into individual functional proteins. This is where 3CLpro comes in - its central part is to cleave these polyproteins at specific sites. Through the cleavage of 3CLpro, nonstructural proteins (nsps) are formed, essential in developing the viral replication-transcription complex. It plays a critical role in the replication and transcription of the virus’ RNA within the host cell (V’kovski et al., 2021; Narwal et al., 2023; Ziebuhr, 2005). For this reason, 3CLpro has been identified as a prime drug target. As a result of the inhibition of 3CLpro, viral polyproteins are prevented from being cleaved into non-structural proteins, effectively inhibiting viral replication and transcription (Mengist et al., 2021). Several efforts are being made to identify and develop inhibitors of 3CLpro, given its importance. Binding to the enzyme’s active site prevents virus replication and transcription, thereby blocking its proteolytic activity.

This study examines the structure of 3CLpro (PDB ID: 7TIA) (Zhang et al., 2020; Forouhar et al., 2022) to outline a comprehensive workflow for identifying potential inhibitors by combining virtual screening, pharmacoinformatics profiling, and molecular dynamics. The crystallographic study of 3CLpro deposited under PDB ID 7TIA facilitated the elucidation of its 3D structure, providing a framework for *in silico* drug discovery (V’kovski et al., 2021). Virtual screening has been widely applied as a cost-effective and efficient strategy to identify potential lead compounds from extensive compound databases (Liu et al., 2022). It has been demonstrated that pharmacoinformatic profiling improves the screening process by prioritizing compounds based on their pharmacokinetic properties, toxicity, and drug likeness, thus facilitating the selection of potential lead compounds (Legare et al., 2022). Molecular dynamics simulations are conducted to gain a deeper understanding of the dynamics of interactions between potential inhibitors and 3CLpro. As a result of these simulations, a greater understanding is gained regarding the stability and flexibility of enzyme-inhibitor complexes, allowing more accurate assessment of the efficacy of inhibitors (Amadei et al., 1993; Alamri et al., 2021; Mody et al., 2021; Khan et al., 2022).

Combining these techniques provides an efficient, comprehensive strategy for identifying potential therapeutic agents, although these techniques contribute independently to drug discovery. As a result of this integrated workflow, promising inhibitors were placed against the 3CLpro of SARS-CoV-2 (PDB ID: 7TIA) in this paper. The findings contribute to the ongoing efforts in combating the COVID-19 pandemic and hold significant potential for advancing therapeutic interventions against SARS-CoV-2. This study aimed to identify potential inhibitors of the enzyme 3CLpro necessary to replicate SARS-CoV-2. The screening of thousands of compounds in various databases was conducted using target-based virtual screening techniques. We will further test these lead compounds *in vitro* and *in vivo*. If successful, they may be developed into a new class of drugs that can combat SARS-CoV-2 and potentially other Coronaviruses in the future.

## 2 Methods and materials

### 2.1 Preparation of ligand library and protein receptor

A total of 7120 compounds were extracted from different databases during the research. OTVA Chemicals (https://www.otavachemicals.com/) provided 862 antiviral compounds, while the Enamine database provided 4201 compounds (https://enamine.net/compound-collections/real-compounds/real-database). Based on PubChem trials, 350 compounds were obtained, 21 compounds were obtained from PubChem records (https://pubchem.ncbi.nlm.nih.gov/docs/covid-19), and 1686 compounds were obtained from Life Chemicals (http://lifechemicals.com/). Using LigPrep (Schrödinger Release 2018), all ligand structures were optimized using the OPLS3e force field, maintaining the specified 3D structure and stereochemistry of the ionization states.

From the RCSB Protein Data Bank, a crystallographic construct of SARS-CoV-2 3CL was obtained with the inhibitor NK01-14, recognizable by the PDB ID 7TIA (Forouhar et al., 2022). Once acquired, this was incorporated into the Maestro suite’s Protein Preparation Wizard (Madhavi Sastry et al., 2013; Schrödinger Release, 2018). A hydrogen atom addition was performed, and adjustments were made to histidine residue protonation states. Additionally, hydrogen bonding patterns were analyzed to determine how water molecules should be removed. An OPLS3e force field was then used to conduct a restrained minimization procedure (Harder et al., 2016). Following the preparation of the protein, the co-crystallized ligand was positioned centrally in a grid box that was created using the grid generation wizard.

### 2.2 Virtual screening and molecular docking

Once prepared, the assembled array of ligands was docked against the designed protein using Glide (Friesner et al., 2006). HTVS mode of Glide facilitates the virtual screening process. An initial procedure was conducted in which compounds were sorted according to the projected binding pocket and ranked based on Glide Scores (GScores). To enhance the precision of the screening, both Standard Precision (SP) and Extra Precision (XP) docking modes were implemented (Friesner et al., 2006).

### 2.3 ADMET and screening analysis

The selected top 22 compounds from XP glide were evaluated and analyzed for their physicochemical and ADMET (Absorption, Distribution, Metabolism, Excretion, and Toxicity) characteristics. The SMILES notations of the significant hits were entered into the ProTox-II server (https://tox-new.charite.de/protox_II/) (Banerjee et al., 2018) to forecast toxicological properties. Furthermore, pharmacokinetic aspects such as absorption, distribution, metabolism, and excretion were evaluated through the SwissADME server (http://www.swissadme.ch/) (Daina et al., 2017). Additionally, BOILED Egg analysis was carried out using the SwissADME web-based tool.

### 2.4 IFD and MMGBSA

Four leading molecules were selected for further analysis based on their Extra Precision (XP) docking scores, molecular interactions with the target protein, and ADMET parameters. When flexible ligand docking is implemented, protein structure can be anticipated using the induced fit docking (IFD) procedure. Compared to rigid ligand-receptor docking commonly encountered in structure-based virtual screening (Sherman et al., 2006a), this method provides a more detailed approach. In addition to the docking evaluation by Glide XP, free binding energy calculations, and ADMET analysis, IFD was performed on the chosen molecules. The process was executed in contrast to the standard protein SARS-CoV-2-3CL, adjusting the van der Waals energy to 0.50 and allowing Prime to generate and refine maximum poses within a radius of five degrees. Induced Fit Docking (IFD) relied on Glide for docking and Prime for refining binding poses following the standard parameters for these modules. A total of 20 poses were generated for each compound (Sherman et al., 2006b). Although this approach does not perfectly mimic biological conditions, it provides valuable insight into the molecule’s stability within a specific binding pocket when presented with various poses and frames.

The Molecular Mechanics Generalized Born Surface Area (MMGBSA) technique was used to compute the relative binding free energy (ΔG bind) for each ligand molecule (Jacobson et al., 2004). This evaluation aimed to determine the binding affinity of the ligand with the receptor, achieved through the Prime module. Using the VSGB 2.0 solvation model and the OPLS3e force field without modification (Brunt et al., 2022), the free binding energy of the top four molecules (hits) has been calculated. Binding affinity is a free energy that includes entropy and enthalpy components. Due to the generalized Born approximation, conformational entropy upon ligand binding was excluded from the MM/GBSA calculations. This provides a quicker but more approximate resolution to the Poisson–Boltzmann equation (Genheden and Ryde, 2015; Wang et al., 2018).

### 2.5 Molecular dynamics simulations

Following the MMGBSA analysis, molecular dynamics simulations were performed on the most promising molecule in order to gain further insight into their stability under biological conditions. Molecular Dynamics (MD) studies can provide insight into the stability of complexes within a solvent system (Bowers et al., 2006). The system was set up in an orthorhombic box with dimensions of 10 Å × 10 Å x 10 Å, derived using the buffer size approach, which also aids in minimizing the box’s volume. The Desmond system builder was used to establish a TIP3P solvent model. In these MD simulation studies, proteins and ions were modeled using the latest OPLS3e force field developed by Schrodinger Inc. (Mark and Nilsson, 2001; Shivakumar et al., 2010; Padhi et al., 2022). Sodium chloride, with Na + as the cation and Cl-as the anion, was incorporated at a concentration of 0.15 M.

A Desmond Molecular Dynamics module was used to operate the system for a runtime of 100 nanoseconds, producing approximately 1000 frames. A molecular dynamic simulation occurred at a stable temperature of 300 K and a pressure of 1 bar, using the NPT ensemble class. The simulation process began after the system model reached a state of relaxation. The molecules were analyzed using MMGBSA (Molecular Mechanics Generalized Born Surface Area) after the molecular dynamic studies had been concluded, and binding free energies were calculated every 10th frame throughout the entire 1000 frames of the simulation (Chaudhary and Dikshit, 2023).

## 3 Results and discussions

### 3.1 Virtual screening and molecular docking

**FIGURE 1:**
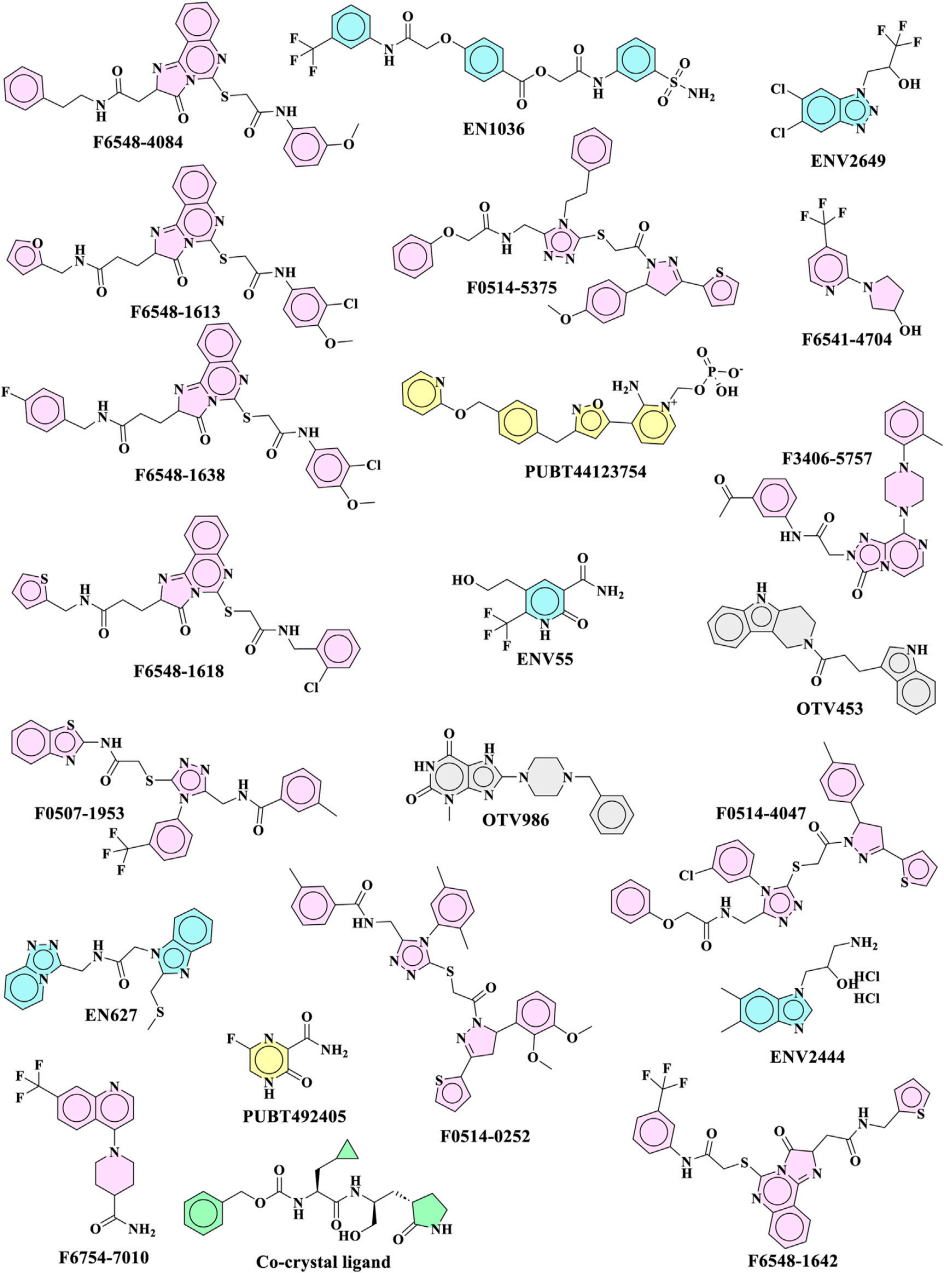
Top 22 molecules received from XP glide docking for further evaluation.

**FIGURE 2:**
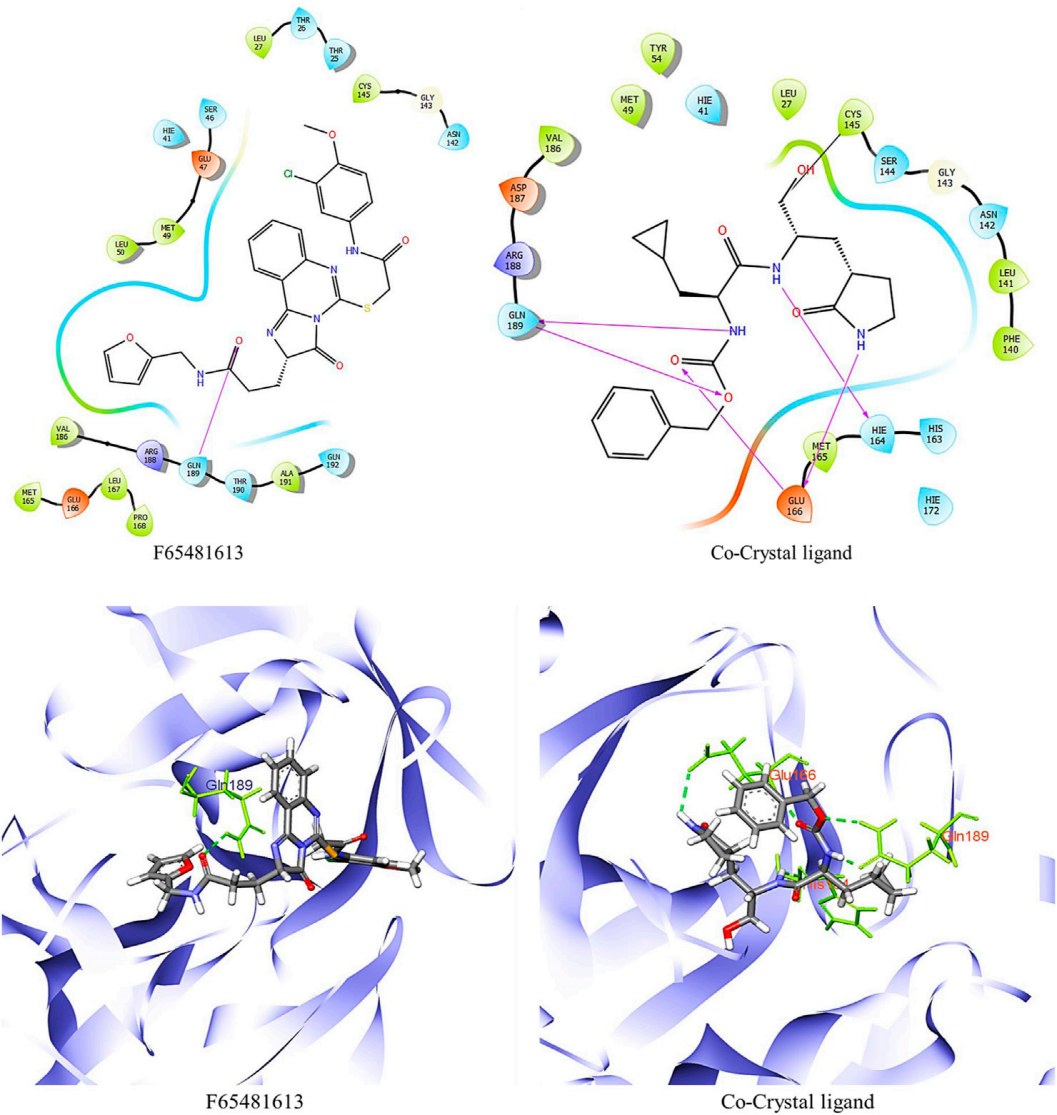
2D and 3D structure of glide docking of compound F6548-1613 and co-crystal ligand.

As part of the High-Throughput Virtual Screening (HTVS) analysis, 5962 compounds from combined libraries were selected. The docking parameters of all molecules can be found in the Supplementary Table S1. As well as docking the selected compounds with the receptor, the native ligand of the receptor was docked separately, allowing for a comparison of the binding affinity between the compounds and the receptor. The initially anticipated binding site was utilized for high-throughput virtual screening (HTVS). In the end, 202 compounds were docked with Standard Precision (SP), and 22 compounds with Extra Precision (XP) docking were docked with SP (Figure 1). The dock cutoff score for SP docking by XP was set at −5.0 kcal/mol based on XP docking outcomes. The glide scores for HTVS and SP ranged from −7.492 to 1.148 Kcal/mol and −5.854 to −2.680 Kcal/mol, respectively, while those for XP ranged from −6.081 to −1.933 Kcal/mol. Most compounds from the Life Chemical library demonstrated better binding affinity than others. The compounds **EN1036, F6548-4084, F6548-1613**, and **PUBT44123754** demonstrated high binding affinity, exceeding the standard ligand. Based on Glide XP scores, 16 molecules showed a better binding affinity than their native ligand (Table 1). The XP glide score for the native ligand was −3.275 kJ/mol, lower than the top four selected compounds. The compounds **F6548-4084, F6548-1613, EN1036,** and **PUBT44123754** recorded docking scores of −5.772, −5.71, −5.78, and −5.003 Kcal/mol, respectively. Among these, compound **F6548-1613** formed a hydrogen bond with Gln189 via the oxygen atom of a carboxamide attached to a furan ring. Distinct hydrophobic interactions are observed with amino acids such as Leu27, Met49, Leu50, Cys145, Met165, Leu167, Pro168, Val186 and Ala191. Polar interactions are also noted with Thr25, Thr26, Hie41, Ser46, Asn142, Gln189, Thr190, and Gln192 (refer to Figure 2). Furthermore, charge interactions, both positive and negative, are identifiable (Table 2). Alongside these, non-bonded interactions with amino acids Hie45, Asn142, Gly143, Cys145, Met165, Glu166, Val186, Arg188, and Gln189 are evident in both the native ligand and compound **F6548-1613** (refer to Figure 2). The same applies to all the selected compounds, **F6548-4084, F6548-1613, EN1036**, and **PUBT44123754**, which showed common interactions with Leu27, Hie41, Met49, Gly143, Asn142, Cys145, Met165 Glu166, Arg188, and Gln189 residues (See Supplementary Figure S1). Induced fit docking (IFD) and MMGBSA calculations were performed alongside the native drug to examine how the selected compounds interact and conform within the protein target’s binding pose.

**TABLE 1:** The Xp glide docking and glide energy scores 22 top molecules.

Compound ID	XP GScore	Glide energy	Compound ID	XP GScore	Glide energy
F6548-4084	−5.772	−54.015	F6548-1618	−3.859	−51.188
EN1036	−5.78	−57.266	F6754-7010	−3.749	−31.828
F6548-1613	−5.71	−59.074	EN627	−3.513	−37.012
PUBT44123754	−5.003	−55.66	F0507-1953	−3.45	−46.265
ENV55	−4.799	−35.654	F0514-0252	−3.446	−42.699
F6548-1638	−4.51	−50.156	co-crystal Ligand	−3.275	−42.6
ENV2649	−4.38	−27.063	F6548-1642	−3.045	−50.945
OTV986	−4.332	−44.094	F0514-4047	−2.726	−50.677
ENV2444	−4.197	−27.86	OTV453	−2.536	−36.798
F6541-4704	−4.129	−18.74	F0514-5375	−2.419	−47.332
PUBT492405	−4.087	−25.072	F3406-5757	−1.933	−47.886

**TABLE 2:** SWISSADME parameters of selected four compounds.

Molecule name	F6548-4084	EN1036	F6548-1613	PUBT44123754	Co-Crystal Ligand
Formula	C29H27N5O4S	C24H20F3N3O7S	C27H24ClN5O5S	C22H21N4O6P	C21H29N3O5
MW	541.62	551.49	566.03	468.4	403.47
#H-bond acceptors	6	11	7	8	29
#H-bond donors	2	3	2	2	6
MR	152.82	127.88	150.09	118.7	110.03
TPSA	139.98	162.27	153.12	157.45	116.76
iLOGP	3.11	2.24	3.58	−1.73	2.56
XLOGP3	3.59	3	3.22	1.68	1.44
WLOGP	2.88	5.02	3.32	2.99	0.49
MLOGP	2.83	1.71	2.16	1.1	0.76
Silicos-IT Log P	4.5	2.42	4.52	1.75	2.12
Consensus Log P	3.38	2.88	3.36	1.16	1.47
ESOL Log S	−5.09	−4.64	−4.98	−3.72	−2.54
ESOL Solubility (mg/mL)	4.45E-03	1.26E-02	5.87E-03	8.84E-02	1.15E+00
ESOL Solubility (mol/L)	8.22E-06	2.28E-05	1.04E-05	1.89E-04	2.86E-03
ESOL Class	Moderately soluble	Moderately soluble	Moderately soluble	Soluble	Soluble
Ali Log S	−6.22	−6.07	−6.11	−4.6	−3.5
Ali Solubility (mg/mL)	3.29E-04	4.67E-04	4.41E-04	1.17E-02	1.28E-01
Ali Solubility (mol/L)	6.08E-07	8.47E-07	7.80E-07	2.51E-05	3.18E-04
Ali Class	Poorly soluble	Poorly soluble	Poorly soluble	Moderately soluble	Soluble
Silicos-IT LogSw	−9.43	−7.88	−9.23	−6.28	−4.62
Silicos-IT Solubility (mg/mL)	2.02E-07	7.19E-06	3.35E-07	2.44E-04	9.75E-03
Silicos-IT Solubility (mol/L)	3.72E-10	1.30E-08	5.92E-10	5.21E-07	2.42E-05
Silicos-IT class	Poorly soluble	Poorly soluble	Poorly soluble	Poorly soluble	Moderately soluble
GI absorption	Low	Low	Low	Low	High
BBB permeant	No	No	No	No	No
Pgp substrate	Yes	No	Yes	Yes	Yes
CYP1A2 inhibitor	Yes	No	Yes	No	No
CYP2C19 inhibitor	Yes	Yes	Yes	Yes	No
CYP2C9 inhibitor	Yes	Yes	Yes	No	No
CYP2D6 inhibitor	Yes	Yes	Yes	No	No
CYP3A4 inhibitor	Yes	No	Yes	Yes	No
log Kp (cm/s)	−7.05	−7.53	−7.47	−7.96	−7.74
Lipinski #violations	1	1	1	0	0
Ghose #violations	2	1	2	0	1
Veber #violations	1	2	2	1	1
Egan #violations	1	1	1	1	1
Muegge #violations	0	2	1	1	1
Bioavailability Score	0.55	0.55	0.55	0.11	0.55
PAINS #alerts	0	0	0	0	0
Brenk #alerts	0	0	0	2	0
Leadlikeness #violations	3	2	2	2	2
Synthetic Accessibility	4.77	3.36	4.58	4.45	4.1

#### 3.1.1 ADMET analysis

A rigorous process was applied to determine potential inhibitors of the SARS-CoV-2 3CLPRO protease. Based on glide docking scores, 22 prominent molecules were identified for further investigation (ADME parameters for all molecules can be found in the Supplementary Table S2). In order to predict the drug-likeness and ADMET (Absorption, Distribution, Metabolism, Excretion, and Toxicity) properties of these ligand molecules, SwissADME and ProTox-II web servers were utilized. Our screening process included applying Lipinski’s Rule of Five to ensure that the screened ligand molecules were suitable for further investigation. Molecules that violated more than one rule were considered unsuitable and therefore excluded. All ligand molecules were evaluated based on their physicochemical properties and drug-like characteristics.

**FIGURE 3:**
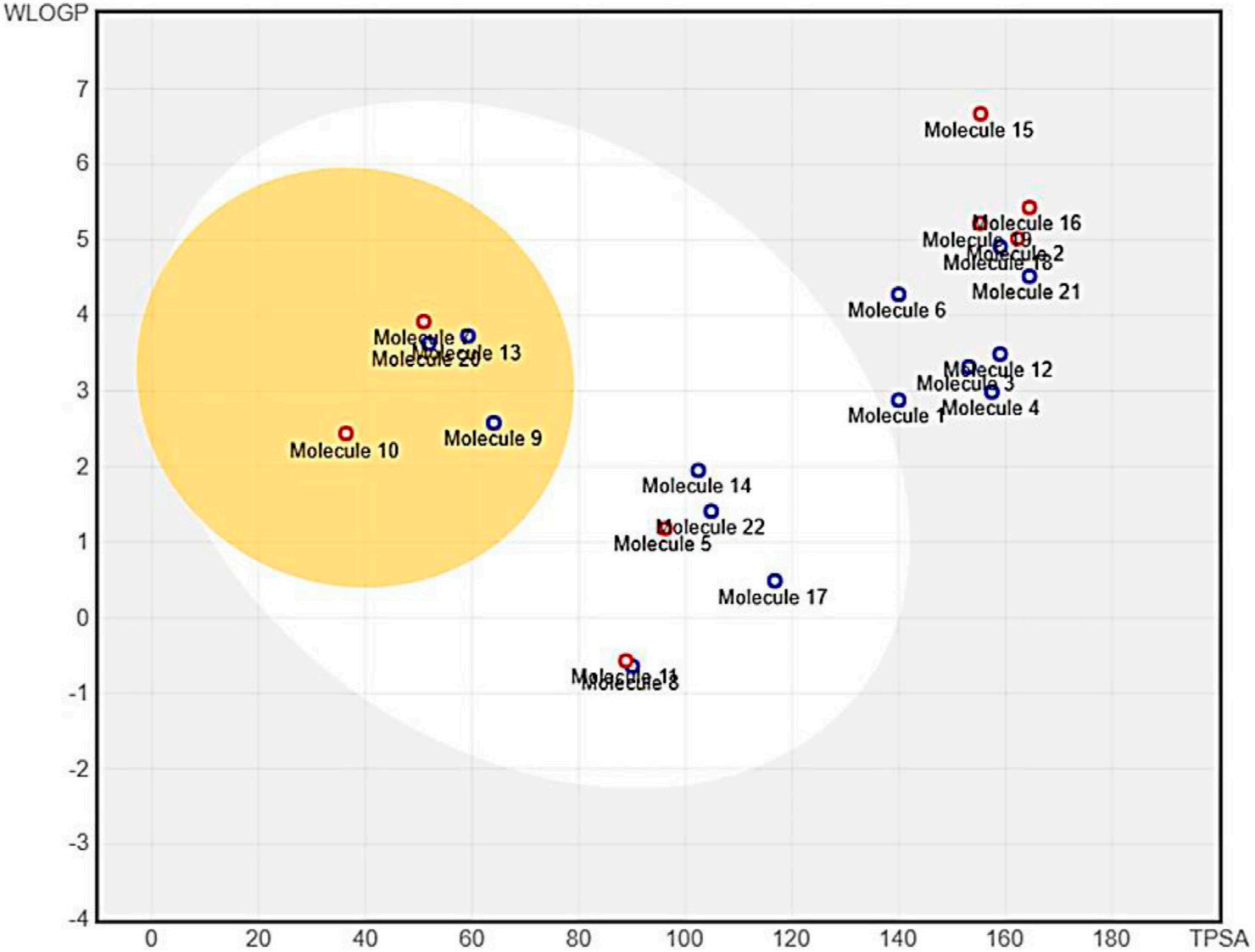
An image from the BOILED-Egg analysis of 22 molecules obtained from the XP Glide procedure.

To provide insight into the potential toxicity of the top four compounds, the ProTox-II webserver was used. The MMGBSA and Induced Fit Docking (IFD) studies were used to determine their ligand-receptor interactions and binding scores. Additionally, we determined the synthetic accessibility (SA) of these compounds, a crucial determinant of drug design since it reflects the ease with which they can be synthesized in the laboratory. It was determined that the SA score ranged from 1 (extremely easy to synthesize) to 10 (very difficult to synthesize). Moreover, we considered the interference of Pan Assay Interference Compounds (PAINS). These molecular structures exhibit promiscuous behavior by reacting in a multitude of assays, often resulting in false-positive results (Table 2). Among the top four compounds, **F6548-1613**, as it is also known, displayed promising results, exhibiting inactivity across multiple parameters, including hepatotoxicity, carcinogenicity, immunotoxicity, mutagenicity, and cytotoxicity. In terms of interactions with various receptors such as AhR, AR, AR-LBD, Aromatase, ER, ER-LBD, PPAR-Gamma, nrf2/ARE, HSE, MMP, p53, ATAD5, compound 2585 is also predicted to be inactive. Notably, the compound demonstrates a high probability of being inactive for interactions with AR-LBD (0.97), AR (0.96), Aromatase (0.96), ER-LBD (0.92), PPAR-Gamma (0.91), HSE (0.88), nrf2/ARE (0.88), and ATAD5 (0.93). Compound **F6548-1613** also demonstrated a high probability of inactivity in interactions with various receptors (Table 2). While all four of the selected compounds displayed more favorable toxicity profiles than standard drugs, compound **PUBT44123754** was noted to exhibit carcinogenicity (Table 3). The compound **F6548-1613** emerged as the most promising candidate, demonstrating excellent toxicity, high inactivity across multiple receptors, and favorable ADMET results. The BOILED-Egg technique assesses the potential of specific phytochemicals to cross the blood-brain barrier and their absorption rates in the gastrointestinal tract. In the BOILED-Egg plot analysis, compounds in the yellow section are more likely to penetrate the blood-brain barrier, while those in the white section are more prone to absorption in the gastrointestinal tract. The white region represents the physicochemical space where molecules have the highest probability of being absorbed by the gastrointestinal tract, and the yellow region (yolk) represents the physicochemical space where molecules have the highest probability of permeating to the brain (Figure 3). It's important to note that the yolk and white areas are not mutually exclusive (Supplementary Figure S2). Molecule 3 (**F6548-1613**) is situated near the white region. This analysis was conducted using the SwissADME web-based tool. These findings suggest compound **F6548-1613** as a potential therapeutic candidate targeting the SARS-CoV-2 3CLPRO protease.

**TABLE 3:** Toxicity parameters of selected four compounds.

Target	Compound ID									
	F6548-4084		EN1036		F6548-1613		PUBT44123754		Co-Crystal ligand	
	Prediction	Probability	Prediction	Probability	Prediction	Probability	Prediction	Probability	Prediction	Probability
Hepatotoxicity	Inactive	0.72	Inactive	0.57	Inactive	0.61	Inactive	0.55	Inactive	0.86
Carcinogenicity	Inactive	0.54	Inactive	0.6	Inactive	0.56	Active	0.52	Inactive	0.68
Immunotoxicity	Inactive	0.98	Inactive	0.92	Inactive	0.98	Inactive	0.98	Inactive	0.99
Mutagenicity	Inactive	0.55	Inactive	0.74	Inactive	0.55	Inactive	0.53	Inactive	0.63
Cytotoxicity	Inactive	0.62	Inactive	0.69	Inactive	0.58	Inactive	0.58	Inactive	0.65
Aryl hydrocarbon Receptor (AhR)	Inactive	0.86	Inactive	0.92	Inactive	0.84	Inactive	0.75	Inactive	0.97
Androgen Receptor (AR)	Inactive	0.94	Inactive	0.94	Inactive	0.96	Inactive	0.95	Inactive	0.97
Androgen Receptor Ligand Binding Domain (AR-LBD)	Inactive	0.96	Inactive	0.96	Inactive	0.97	Inactive	0.98	Inactive	0.99
Aromatase	Inactive	0.93	Inactive	0.96	Inactive	0.96	Inactive	0.89	Inactive	0.98
Estrogen Receptor Alpha (ER)	Inactive	0.87	Inactive	0.94	Inactive	0.86	Inactive	0.87	Inactive	0.9
Estrogen Receptor Ligand Binding Domain (ER-LBD)	Inactive	0.94	Inactive	0.99	Inactive	0.92	Inactive	0.96	Inactive	0.96
Peroxisome Proliferator Activated Receptor Gamma (PPAR-Gamma)	Inactive	0.87	Inactive	0.92	Inactive	0.91	Inactive	0.95	Inactive	0.98
Nuclear factor (erythroid-derived 2)-like 2/antioxidant responsive element (nrf2/ARE)	Inactive	0.91	Inactive	0.98	Inactive	0.88	Inactive	0.91	Inactive	0.97
Heat shock factor response element (HSE)	Inactive	0.91	Inactive	0.98	Inactive	0.88	Inactive	0.91	Inactive	0.97
Mitochondrial Membrane Potential (MMP)	Inactive	0.77	Inactive	0.82	Inactive	0.78	Inactive	0.79	Inactive	0.95
Phosphoprotein (Tumor Supressor) p53	Inactive	0.83	Inactive	0.93	Inactive	0.82	Inactive	0.87	Inactive	0.92
ATPase family AAA domain-containing protein 5 (ATAD5)	Inactive	0.91	Inactive	0.98	Inactive	0.93	Inactive	0.93	Inactive	0.95

### 3.2 IFD and MMGBSA

**FIGURE 4:**
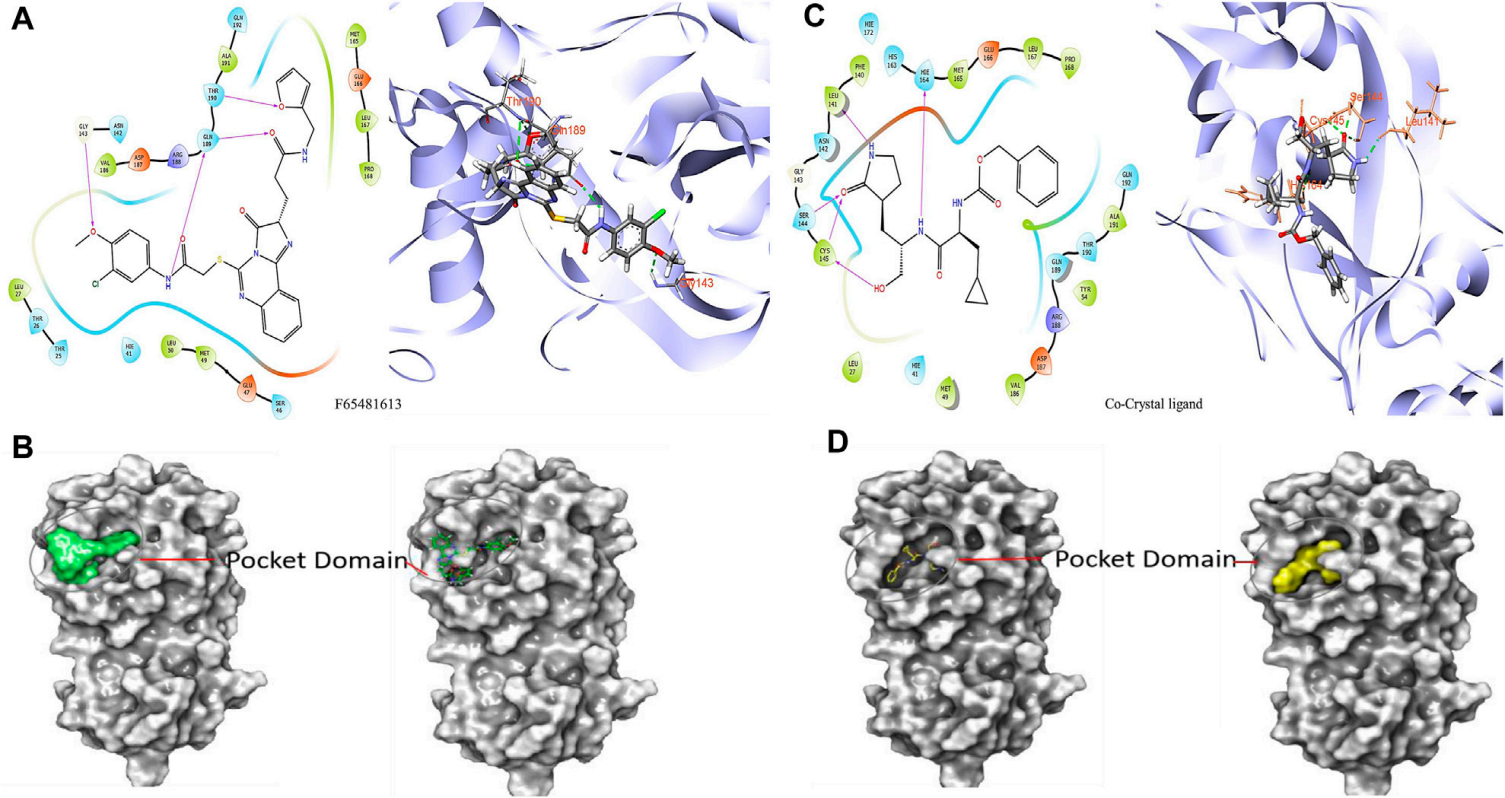
Images of molecules derived from Induced Fit Docking (IFD) in both 2D and 3D forms: **(A, B)** Depict the compound **F6548-1613** and its surface structure. **(C, D)** Show the Co-Crystal Ligand and its surface structure.

It was determined that the protein exhibits substantial conformational variation, which was analyzed through the Induced Fit Docking (IFD) strategy, utilizing GLIDE-XP results. To understand the potential binding conformational attributes of the top four inhibitors, **EN1036, F6548-4084, F6548-1613**, and **PUBT44123754**, IFD computations were performed (see Table 4; Figure 4 and See Supplementary Figure S4). Figure 4 illustrates how the inhibitor docks within the catalytic pockets of the 7TIA enzyme. Compound **F6548-1613** displayed the most potent binding affinity from the shortlisted compounds, predicting 16 different poses with 3CLpro. The primary pose had the highest IFD score of −675.26 Kcal/mol, as indicated in Table 4. According to the investigation, pose 13, with an Induced Fit Docking (IFD) score of −668.47 Kcal/mol, exhibits the highest number of advantageous bondings and non-bondings with the primary protease. **F6548-1613** established four hydrogen bonds with the protein. These included interactions with the 4th oxygen group of the benzene ring by Gly143, the NH group of the carboxamide substituted benzene ring by Gln189 with two groups, the NH group of the carboxamide substituted benzene ring, and the oxygen atom of the carboxamide linked with the furan ring, and the 3rd oxygen atom of the furan ring with the Thr190 residue. These interactions are illustrated in both 2D and 3D diagrams (refer to Figure 4). The IFD scores and 3D interactions can be found in Table 4 and Figure 4. Compound **F6548-1613** was observed to interact with the same amino acid residue loops as the native ligand (co-crystal ligand), comprising residues like Met165, Glu166, Leu167, Pro168, Val186, Asp187, Arg188, Gln189, Thr190, Ala191, and Gln192. Furthermore, specific amino acid residues like Hie41, Met49, and Tyr210 showed common interaction in Compound **F6548-1613** and standard drugs.

**TABLE 4:** The docking scores, IFD, and MM-GBSA of top 4 molecules.

Compound ID	XP GScore	Glide energy	IFD	MMGBSA	Prime energy
F6548-4084	−5.772	−54.015	−672.51	−46.88	−12557.54
EN1036	−5.78	−57.266	−671.66	−64.68	−12545.35
F6548-1613	−5.71	−59.074	−673.34	−65.72	−12556.57
PUBT44123754	−5.003	−55.66	−675.26	−59.18	−12582.05
co-crystal Ligand	−3.275	−42.6	−669.98	−38.26	−12512.93

Compound **F6548-1613**, one of the top hits, displayed superior binding free energies compared to standard compounds, exhibiting the highest binding energy of −65.72 kcal/mol when interacting with 7TIA. A native ligand, on the other hand, showed ΔG binding energies of −58.59, −63.29, and −38.26 kcal/mol. The binding energies of GbindHbond, GbindvdW, GbindLipo, and GbindCoulomb were significant (see Supplementary Table S4). Due to its optimal balance of several types of interactions, **F6548-1613** emerges as the most effective candidate for targeting SARS-CoV-2 3CLPRO protease. This compound’s MMGBSA dG Bind vdW score is the lowest (−54.55), indicating robust van der Waals interactions, contributing significantly to its high binding affinity. Despite Compound **EN1036** having the lowest GbindCoulomb score, which suggests potent Coulombic or electrostatic interactions, Compound **F6548-1613** still demonstrated the highest overall binding affinity based on a summation of its various interactions.

Furthermore, Compound **F6548-1613** displayed the lowest GbindLipo score, indicating the presence of lipophilic or hydrophobic interactions that enhance protein-ligand binding and complex stability. Again, its GbindPacking score was among the lowest, indicating superior packing interactions and a well-fitted ligand within the protein’s binding pocket. However, despite **F6548-1613** and **PUBT44123754** showing relatively less negative GbindHbond scores, suggesting fewer or weaker hydrogen bonds, **F6548-1613’s** binding affinity remained superior. This dominance can be attributed to the combination of all its interaction types. GbindSolvGB (Solvation Energy) score, a measure of the free energy of solvation, indicates how favorably a ligand is solvated before entering the binding pocket. In this regard, **F6548-1613** demonstrated a highly positive score (28.92), denoting a favorable solvation energy. All compounds, including **F6548-1613,** displayed positive GbindCovalent scores, a trait often associated with the absence of covalent bonds or the presence of destabilizing covalent interactions.

Nevertheless, **F6548-1613** had the lowest positive value, suggesting the least destabilizing influence from potential covalent interactions. Molecular Dynamics (MD) was performed to analyze the stability of **F6548-1613** within the protein target’s binding pose, providing insight into the molecule’s stability in the binding pocket against 3CL protease. This additional layer of analysis further substantiates **F6548-1613’s** potential as an effective inhibitor of the SARS-CoV-2 3CLPRO protease.

### 3.3 Molecular dynamics

**FIGURE 5:**
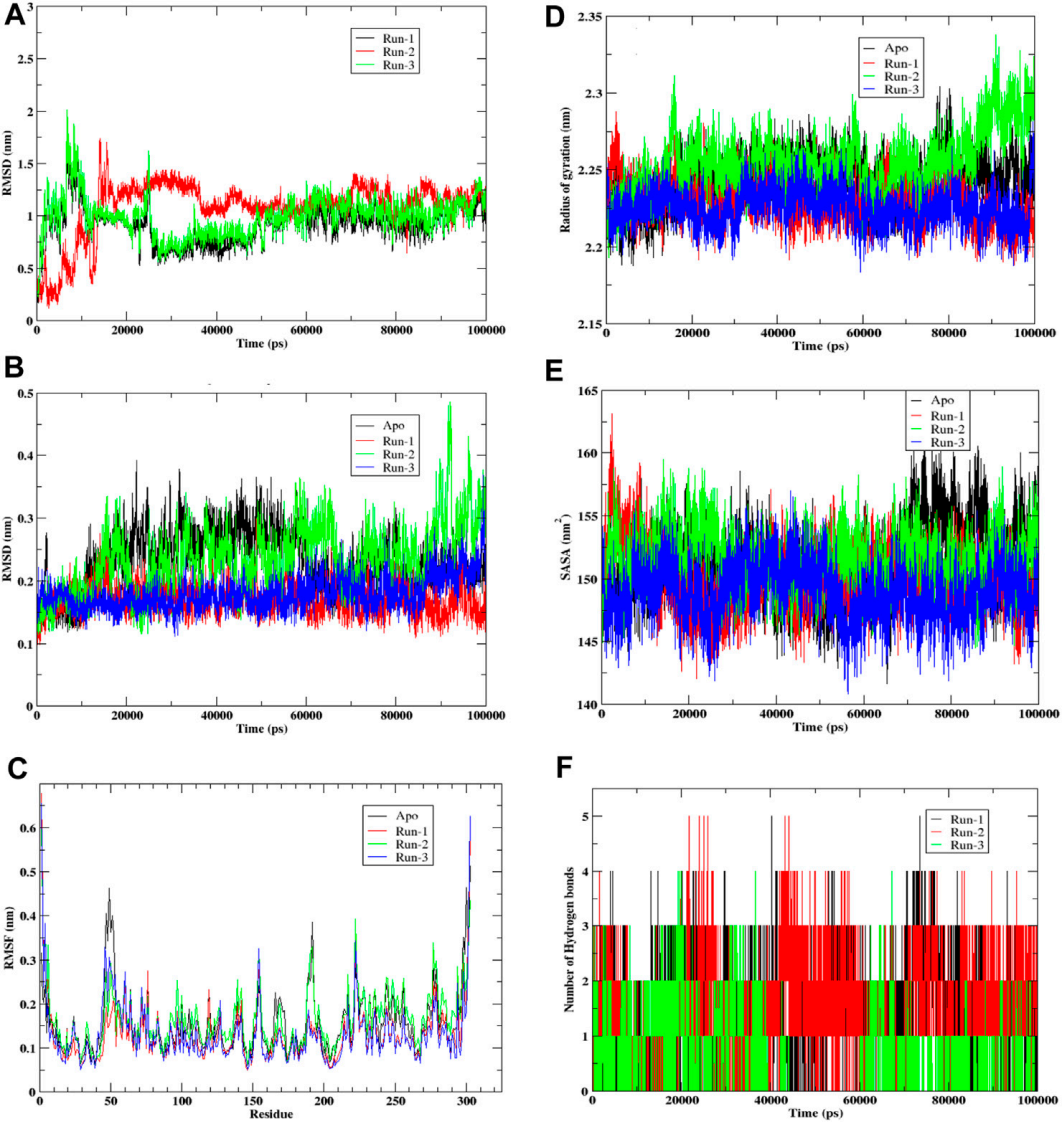
Molecular Dynamic simulation of hit compound **F6548-1613** as triplicate. **(A)** The root mean square deviation (RMSD) variation for proteins and ligands over time. **(B)** The root mean square deviation (RMSD) variation for APO proteins. **(C)** Protein fluctuations and a ligand were analyzed via Root Mean Square Fluctuation (RMSF) for each residue. **(D)** Graph illustrates the radius of gyration for a protein. **(E)** Graph illustrates the Solvent Accessible Surface Area (SASA) of the protein, a metric measured in square angstroms (Å^2^) that reflects the surface area accessible to a solvent, typically water in biological systems. **(F)** A histogram that shows No. of Hydrohen bonding.

The presented graph (Figure 5A) depicts Root Mean Square Deviation (RMSD), a commonly employed statistic for evaluating disparities in atomic positions across various time points. The x-axis corresponds to the time in picoseconds (ps), while the y-axis displays RMSD values in nanometers (nm). The graph exhibits three distinctive simulation runs—Run-1 in black, Run-2 in red, and Run-3 in green. Observations from the graph indicate that RMSD readings peak during the initial 20,000 picoseconds (or 20 nanoseconds) for all three runs, suggesting potential initial fitting adjustments. Following this phase, each run’s RMSD tends to stabilize, fluctuating around specific values. This stabilization implies that the ligand’s positioning relative to the protein reaches a relatively constant state, with occasional deviations possibly arising from interactions within the simulation environment or conformational changes. Notably, Run-3 (green) displays the lowest average RMSD, indicating that, in contrast to the preceding runs, the ligand in this simulation maintains a more consistent position or conformation throughout. In comparison, runs 1 (black) and 2 (red) exhibit similar patterns with slightly higher RMSD values, implying increased conformational flexibility. This flexibility may be attributed to the ligand’s free end, lacking hydrogen bond formation with amino acid residues. Despite the initial instability observed in all three simulation runs, they eventually establish a dynamic equilibrium, where the ligand’s position fluctuates around a relatively constant value, as illustrated in the RMSD plot.

Graph (Figure 5B) visually represents the Root Mean Square Deviation (RMSD) of C-alpha atoms over time, obtained through least squares fitting to a protein in a molecular dynamics simulation. The y-axis reflects RMSD in nanometers (nm), while the x-axis corresponds to time in picoseconds (ps). The graph encompasses four distinct traces: the Apo form in black, the control run without any ligand; Run-1 in red; Run-2 in green; and Run-3 in blue, representing three simulation runs for the protein-ligand complex. Examining the graph, it is evident that the RMSD values for each run commence at relatively low levels, indicating minimal initial deviation of the protein’s C-alpha atoms from the reference structure, which is the starting point of the simulation. As time progresses, the RMSD values undergo shifts yet consistently maintain within a narrow range (approximately 0.1–0.4 nm). This suggests a lack of significant conformational changes in the protein structures throughout these simulation runs. Notably, the black trace representing the Apo form exhibits the least fluctuation, implying that the protein remains relatively stable in its original state or when devoid of any ligand.

In contrast, Runs 1, 2, and 3 display more significant fluctuations attributed to the presence of the ligand. Despite this increased movement, the variations observed in these runs remain confined within a limited range, suggesting the absence of substantial structural alterations. In summary, the RMSD graph provides insights into the stability of the protein’s secondary structure during the simulation. The Apo run demonstrates minimal deviation, while the other runs, influenced by interactions with the ligand, exhibit increased movement within a constrained range. This overall pattern indicates that the protein maintains structural stability throughout the simulation, even in the presence of a ligand.

The provided graph (Figure 5C) illustrates a protein’s Root Mean Square Fluctuation (RMSF), portraying the flexibility of specific residues during a simulation. Higher RMSF values indicate increased flexibility or mobility of these residues within the protein structure. The y-axis represents RMSF values in nanometers (nm), showing the extent of fluctuation for each residue. At the same time, the x-axis lists the residues of the protein sequence from the N-terminus to the C-terminus. The data from four distinct runs are depicted in the plot: Run-1 (red), Run-2 (green), Run-3 (blue), and Apo (black), with the Apo run representing the unbound or native state of the protein without a ligand. Notably, the Apo run serves as a baseline, displaying consistent fluctuation across residues. Across all runs, a similar fluctuation pattern is observed, suggesting that the overall flexibility of the protein remains relatively constant throughout the simulation runs. Peaks in the graph represent residues that exhibit higher flexibility, and these peaks are consistent across all runs, indicating inherent flexibility in specific regions of the protein. Most residues demonstrate moderate fluctuations ranging between 0.1 and 0.3 nm, with fluctuations typically staying below 0.4 nm. Towards the end of the residue sequence, prominent peaks emerge where RMSF values are notably higher. This is attributed to relatively lesser interaction in the terminal residues. Notably, residues such as Tyr154 and Arg222 consistently show maximum fluctuation across all three runs. Additionally, a peak at Leu50 in the Apo form, which is not as pronounced in the other runs, suggests that this residue is stabilized by adding the ligand in the binding pocket. While small differences exist between runs, the overall patterns are similar, indicating that the ligand or the conditions tested do not significantly alter the protein’s flexibility at the residue level. This suggests that the protein maintains a consistent level of flexibility across various simulation conditions.

Graph (Figure 5D) illustrates the radius of gyration for a protein, indicating how its mass is distributed from its center of mass and reflecting the molecule’s compactness. The x-axis represents time in picoseconds (ps), while the y-axis displays the radius of gyration in nanometers (nm). The four simulation runs represented by lines are Apo (black), Run-1 (red), Run-2 (green), and Run-3 (blue). Throughout all simulation runs, the radius of gyration exhibits variations within a relatively confined range, approximately 2.15 nm–2.35 nm. These variations suggest changes in the protein structure’s compactness over time, albeit within a relatively short range. Notably, the average radius of gyration for the Apo form appears to be the lowest, indicating that the protein is more compact in its unbound or reference state. Runs 1, 2, and 3 show comparable trends as the gyration radius gradually increases. This suggests that these proteins experience a loss of compactness, potentially due to unfolding or conformational changes induced by the interacting ligand. The overall plot indicates that the protein structures in simulation undergo some conformational change, although this change is not notably high. In summary, the radius of the gyration graph provides insights into the dynamic changes in the protein’s compactness over time in different simulation conditions. While there is observable variation, the alterations remain within a limited range, and the protein structures experience some degree of conformational change, particularly when interacting with the ligand.

Graph (Figure 5E) illustrates the Solvent Accessible Surface Area (SASA) of the protein, a metric measured in square angstroms (Å^2^) that reflects the surface area accessible to a solvent, typically water in biological systems. The y-axis represents SASA values, while the x-axis depicts time in picoseconds (ps). The graph encompasses four traces corresponding to different simulation runs: Apo (black), Run-1 (red), Run-2 (green), and Run-3 (blue). The dynamic nature of the protein’s conformational changes is evident as SASA varies over time for each run, reflecting alterations in the solvent-exposed area of the molecule. The Apo form (black) notably exhibits the highest average SASA, indicating a greater average exposed surface area when the protein is unbound.

In contrast, the Runs 1, 2, and 3 SASA values consistently appear somewhat lower than the Apo form, suggesting a more compact or less exposed structure in these scenarios. The similar fluctuation patterns across these runs imply a comparable behavior in terms of solvent accessibility. Despite dynamic fluctuations, the SASA plot reveals no sharp rises or falls for any of the runs, indicating that significant unfolding or refolding does not occur during the simulation period.

Furthermore, the SASA for each run consistently remains lower than that of the Apo form, implying an increase in the folding of the protein upon interaction with the ligand. In summary, the SASA graph provides a comprehensive view of the protein’s dynamic behavior, suggesting that while there are variations in solvent accessibility over time, the protein’s overall structure remains stable. The protein undergoes conformational fluctuations without experiencing substantial unfolding or refolding during the simulated conditions.

Graph (Figure 5F) illustrates the dynamic evolution of hydrogen bond formation over time in picoseconds (ps) across three distinct runs (Run-1, Run-2, and Run-3) involving compound F6548-1613 complexed with the target protein 3CLpro. The y-axis quantifies the number of hydrogen bonds formed between the protein and ligand, while the x-axis represents the temporal progression in picoseconds. Each run is differentiated by black, red, and green bars, corresponding to Run-1, Run-2, and Run-3. Examining the graph, it is evident that the total count of hydrogen bonds for each run exhibits notable variability over time, showcasing irregular fluctuations in bond numbers. The data portray a seemingly erratic pattern with no discernible trend or pattern across the simulation duration for any run. This suggests a certain degree of randomness in both the creation and disruption of hydrogen bonds, indicating continuous and variable occurrences of bond formation and breaking.

Moreover, the absence of a clear distinction between the runs implies that the conditions or factors influencing hydrogen bond formation were likely consistent across all three experimental runs. Exploring potential reasons for the absence of significant differences between each run could provide valuable insights into the intrinsic variability of the observed events. This detailed analysis could contribute to a deeper understanding of hydrogen bond dynamics’ continuous and dynamic nature under the specified experimental conditions.

## 4 Conclusion

This study aimed to identify effective inhibitors against the SARS-CoV-2 3CL protease through an in-depth virtual screening process. A collection of 5962 compounds underwent rigorous evaluations, including standard and extra precision docking, high-throughput virtual screening (HTVS), and hit-to-lead optimization. The compounds were compared to the co-crystal ligand NK01-14 inhibitor. Among the four selected compounds, namely **EN1036, F6548-4084, F6548-1613**, and **PUBT44123754**, they all demonstrated solid binding affinities ranging from −5.003 to −5.772 kcal/mol and exhibited superior IFD scores ranging from −671.66 to −675.26 kcal/mol. Remarkably, these results surpassed the performance of the co-crystallized ligand. Notably, compound **F6548-1613** exhibited superior interactions and binding poses, showcasing its potential as a lead compound. Molecular dynamics simulations confirmed the stability of compound **F6548-1613**, with consistent positioning observed in Run-3. The compound **F6548-1613** displayed notable interactions with key amino acid residues, particularly Thr26 and His41, surpassing the reference inhibitor NK01-14. Additionally, compound **F6548-1613** demonstrated excellent toxicity profiles, inactivity across multiple receptors, and favorable ADMET results, making it a promising candidate for further development. The BOILED-Egg technique supported its synthetic accessibility. In conclusion, this study provides comprehensive insights into the selection and optimization of potential inhibitors for the SARS-CoV-2 3CL protease, with compound **F6548-1613** emerging as a standout candidate. Molecular dynamics simulations reinforced its stability and interactions, emphasizing its potential as an effective inhibitor. Further experimental validation through *in vitro* and *in vivo* studies is recommended to confirm its antiviral efficacy.

## Data Availability

The original contributions presented in the study are included in the article/Supplementary Material, further inquiries can be directed to the corresponding author.
