# Supplementary material for: Integrating virtual screening, pharmacoinformatics profiling, and molecular dynamics: identification of promising inhibitors targeting 3CLpro of SARS-CoV-2
Source: Front Mol Biosci. 2024 Mar 7;10:1306179. doi: 10.3389/fmolb.2023.1306179 (PMC10956415; doi:10.3389/fmolb.2023.1306179)
Supplement: Supplementary file 3 [file Table3.docx]

**Table S3.** An image from the BOILED-Egg analysis of 22 molecules obtained from the XP Glide procedure.

| Molecule name | Molecule name | Canonical SMILES |
| --- | --- | --- |
| Molecule 1 | F6548-4084 | COc1cccc(c1)NC(=O)CSc1nc2ccccc2c2=NC(C(=O)n12)CC(=O)NCCc1ccccc1 |
| Molecule 2 | EN1036 | O=C(Nc1cccc(c1)C(F)(F)F)COc1ccc(cc1)C(=O)OCC(=O)Nc1cccc(c1)S(=O)(=O)N |
| Molecule 3 | F6548-1613 | COc1ccc(cc1Cl)NC(=O)CSc1nc2ccccc2c2=NC(C(=O)n12)CCC(=O)NCc1ccco1 |
| Molecule 4 | PUBT44123754 | Nc1[n+](cccc1c1onc(c1)Cc1ccc(cc1)COc1ccccn1)CO[P@](=O)(O)[O-] |
| Molecule 5 | ENV55 | OCCc1cc(C(=O)N)c(=O)[nH]c1C(F)(F)F |
| Molecule 6 | F6548-1638 | COc1ccc(cc1Cl)NC(=O)CSc1nc2ccccc2c2=NC(C(=O)n12)CCC(=O)NCc1ccc(cc1)F |
| Molecule 7 | ENV2649 | OC(C(F)(F)F)Cn1nnc2c1cc(Cl)c(c2)Cl |
| Molecule 8 | OTV986 | O=c1[nH]c(=O)n(c2c1[nH]c(n2)N1CCN(CC1)Cc1ccccc1)C |
| Molecule 9 | ENV2444 | NCC(Cn1cnc2c1cc(C)c(c2)C)O.Cl.Cl |
| Molecule 10 | F6541-4704 | OC1CCN(C1)c1nccc(c1)C(F)(F)F |
| Molecule 11 | PUBT492405 | Fc1c[nH]c(=O)c(n1)C(=O)N |
| Molecule 12 | F6548-1618 | O=C(CSc1nc2ccccc2c2=NC(C(=O)n12)CCC(=O)NCc1cccs1)NCc1ccccc1Cl |
| Molecule 13 | F6754-7010 | NC(=O)C1CCN(CC1)c1ccnc2c1ccc(c2)C(F)(F)F |
| Molecule 14 | EN627 | CSCc1nc2c(n1CC(=O)NCc1nnc3n1cccc3)cccc2 |
| Molecule 15 | F0507-1953 | O=C(Nc1nc2c(s1)cccc2)CSc1nnc(n1c1cccc(c1)C(F)(F)F)CNC(=O)c1cccc(c1)C |
| Molecule 16 | F0514-0252 | COc1cccc(c1OC)C1CC(=NN1C(=O)CSc1nnc(n1c1cc(C)ccc1C)CNC(=O)c1cccc(c1)C)c1cccs1 |
| Molecule 17 | CO-Crystal ligand | OC[C@@H](NC(=O)[C@@H](NC(=O)OCc1ccccc1)CC1CC1)C[C@@H]1CCNC1=O |
| Molecule 18 | F6548-1642 | O=C(Nc1cccc(c1)C(F)(F)F)CSc1nc2ccccc2c2=NC(C(=O)n12)CC(=O)NCc1cccs1 |
| Molecule 19 | F0514-4047 | O=C(NCc1nnc(n1c1cccc(c1)Cl)SCC(=O)N1N=C(CC1c1ccc(cc1)C)c1cccs1)COc1ccccc1 |
| Molecule 20 | OTV453 | O=C(N1CCc2c(C1)c1ccccc1[nH]2)CCc1c[nH]c2c1cccc2 |
| Molecule 21 | F0514-5375 | COc1ccc(cc1)C1CC(=NN1C(=O)CSc1nnc(n1CCc1ccccc1)CNC(=O)COc1ccccc1)c1cccs1 |
| Molecule 22 | F3406-5757 | O=C(Cn1nc2n(c1=O)ccnc2N1CCN(CC1)c1ccccc1C)Nc1cccc(c1)C(=O)C |
