# Supplementary material for: Integrating virtual screening, pharmacoinformatics profiling, and molecular dynamics: identification of promising inhibitors targeting 3CLpro of SARS-CoV-2
Source: Front Mol Biosci. 2024 Mar 7;10:1306179. doi: 10.3389/fmolb.2023.1306179 (PMC10956415; doi:10.3389/fmolb.2023.1306179)
Supplement: Supplementary file 5 [file DataSheet1.docx]

**Supplementary File**

**Integrating Virtual Screening, Pharmacoinformatics Profiling, and Molecular Dynamics: Identification of Promising Inhibitors Targeting 3CLpro of SARS-CoV-2**


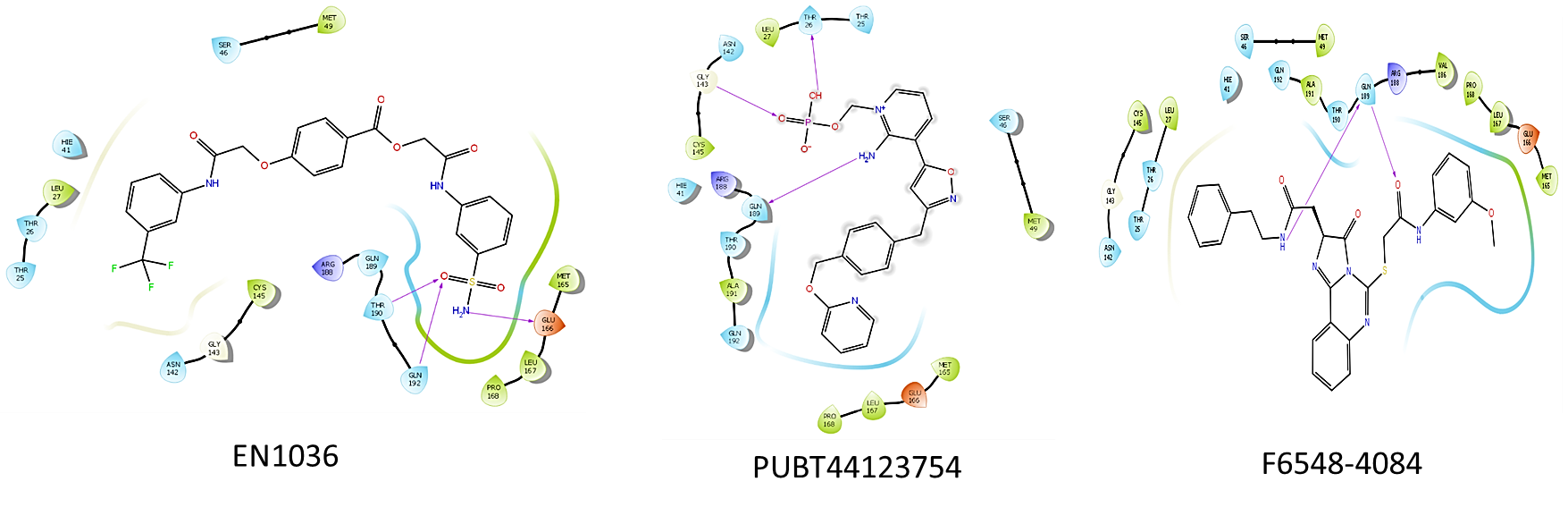


**Fig.S1.** 2D structure of glide docking of compound EN1036, PUBT44123754 and F6548-4084.


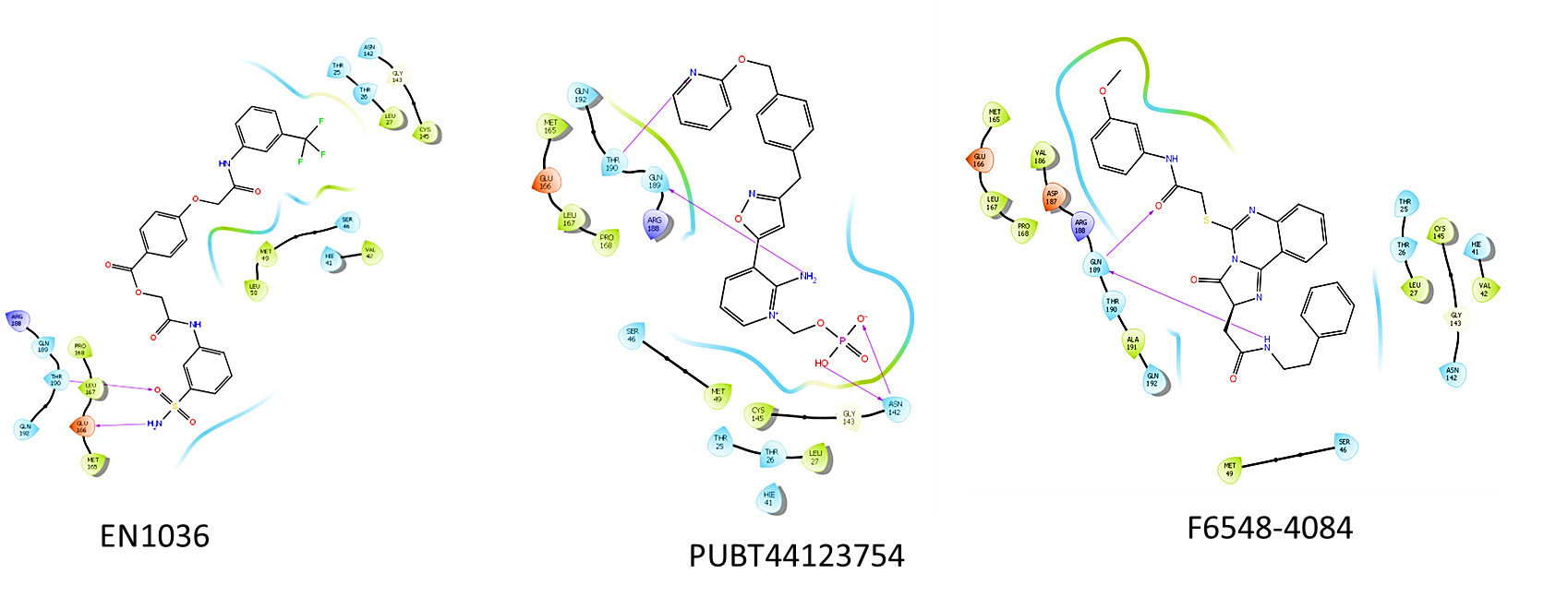


**Fig.S2.** Images of molecules derived from Induced Fit Docking (IFD) in 2D of compound EN1036, PUBT44123754 and F6548-4084.
